# Why should a 5q spinal muscular atrophy neonatal screening program be started?

**DOI:** 10.1055/s-0044-1791201

**Published:** 2024-10-13

**Authors:** Michele Michelin Becker, Flávia Nardes, Tamara Dangouloff, Laurent Servais, Alexandra Prufer de Queiroz Campos Araujo, Juliana Gurgel-Giannetti

**Affiliations:** 1 Hospital de Clínicas de Porto Alegre, Unidade de Neurologia Pediátrica, Departamento de Pediatria, Porto Alegre RS, Brazil.; 2 Universidade Federal do Rio de Janeiro, Instituto de Puericultura e Pediatria Martagão Gesteira, Departamento de Pediatria, Rio de Janeiro RJ, Brazil.; 3 Université de Liège, Centre Hospitalier Universitaire de Liège, Centre de Référence des Maladies Neuromusculaires, Service de Pédiatrie, Liège, Belgium.; 4 University of Oxford, MDUK Neuromuscular Centre, Oxford, United Kingdom.; 5 Universidade Federal de Minas Gerais, Faculdade de Medicina, Departamento de Pediatria, Belo Horizonte MG, Brazil.

**Keywords:** Muscular Atrophy, Spinal, Neonatal Screening, Early Medical Intervention, Atrofia Muscular Espinhal, Triagem Neonatal, Intervenção Médica Precoce

## Abstract

Spinal muscular atrophy (SMA) is a genetic neuromuscular progressive disorder that is currently treatable. The sooner the disease-modifying therapies are started, the better the prognosis. Newborn screening for SMA, which is already performed in many countries, has been scheduled to begin in the near future. The development of a well-organized program is paramount to achieve favorable outcomes for the child who is born with the disease and for the costs involved in health care. We herein present a review paper hoping to point out that SMA neonatal screening is urgent and will not increase the cost of its care.

## Resumo

A atrofia muscular espinhal (AME) é uma doença genética neuromuscular progressiva tratável atualmente. Quanto antes o tratamento com as terapias modificadoras da doença for iniciado, melhor será o prognóstico. A triagem neonatal para a AME, já implementada em vários países, está programada para iniciar no Brasil em um futuro próximo. O desenvolvimento de um programa bem organizado é fundamental para que se alcance um resultado favorável para as crianças que nascem com a doença e para os custos envolvidos nos cuidados de saúde. Apresentamos um artigo de revisão na esperança de enfatizar que a triagem neonatal para a AME é urgente e não aumentará os custos relacionados aos cuidados.

## INTRODUCTION

Newborn screening (NBS) is a set of preventive actions aimed at diagnosing diseases that lead to severe deficits if left untreated, preferably at a presymptomatic stage. It goes far beyond a diagnostic test: it is a very well-structured program that includes timely diagnosis, timely treatment, and specialized follow-up. 1 The early detection of treatable congenital diseases though NBS prevents severe disability and death, which is a great achievement for those who are affected and their families. 2 Successful NBS requires the diagnosis and initiation of treatment in the first days or weeks of life, before the onset of clinical manifestations,. 3

Over the last decade, several therapeutic approaches have been developed that have changed the natural history of spinal muscular atrophy (SMA). Currently, there are three medications for SMA approved by the United States Food and Drug Administration (FDA) and in several countries (nusinsersen, onasemnogene abeparvovec, and risdiplam), with the best results being obtained with early treatment. Unfortunately, there is no evidence that patients treated after the onset of symptoms can develop normally, which reinforces the importance of early diagnosis and treatment, ideally in the presymptomatic phase. 4

Recently, many pilot studies on NBS for SMA have been launched worldwide, and some countries have already included SMA in the NBS. 5 The expansion of the NBS in Brazil was recently approved, which will include SMA in the forthcoming future.

The current review summarizes information about this disease and about the impact of early diagnosis to benefit from the best therapeutic window, which results in the best prognostic scenario. Cost studies 1 have shown that, through this initiative, the costs are rather limited than expanded. The authors of the present review provide their opinion and reflect on the obstacles that could be anticipated and need to be previously planned for, to establish the program in a large and uneven country such as Brazil.

## SPINAL MUSCULAR ATROPHY

### The disease is rare, but it is the main genetic cause of infant mortality

Spinal muscular atrophy linked to chromosome 5q (SMA 5q) is an autosomal recessive disease caused by mutations on chromosome 5 in the *SMN1* gene that lead to reduced expression of the survival motor neuron (SMN) protein. This causes progressive degeneration of alpha-motor neurons in the spinal cord and brainstem, with muscle atrophy and weakness of the limb, trunk, and the bulbar and respiratory muscles. The worldwide incidence of SMA is of ∼ 1 in 14,300 live births. 6 Among the general population, the frequency of carriers of the *SMN1* gene mutation ranges from 1 in 38 to 1 in 72, with a panethnic mean of 1 in 54. 6 7 8 9 Although SMA 5q is a rare disease, it is the main genetic cause of infant mortality and the second most frequent neuromuscular disease in childhood. 10 The high prevalence of carriers in the population means that most of the time there is no history cases of the disease in the family, which would accelerate the diagnosis.

### Genetics in SMA is well established and known

About 95% of patients with SMA 5q have homozygous deletions of both exons 7 and 8 or only of exon 7 of the *SMN1* gene. About 5% of patients carry an SMN1 point mutation and a deletion in the other *SMN1* allele, or, rarely, biallelic small-point mutations in any of the *SMN1* exons. De novo mutations occur at a relatively high rate (∼ 2%) due to the instability of this 5q region. 11 In a Brazilian cohort study with 450 patients with SMA, Mendonça et al. 11 found that 89.3% of the patients presented homozygous exon 7- *SMN1* deletion, and 10.7% were compound heterozygous for the common deletion in one allele and a point mutation in the other allele. The *SMN2* gene copy number is one of the important factors of phenotypic severity and has an inverse correlation in SMA patients. 8 Most patients with type-1 SMA (80%) have 1 or 2 copies of *SMN2* , 78% of those with type-2 SMA have 3 copies of *SMN2* , and 93% of the patients with type-3 SMA have 3 or 4 copies of *SMN2* . 12

### Pathophysiology begins in utero, before the clinical manifestations

Skeletal muscle atrophy and weakness are caused by disruption of the entire motor unit. The initial stages of the disease are characterized by impairments and slowing in development that affect the motor neuron (cell body, axon, excitatory synaptic inputs in the neuromuscular junction, axon radial growth, and myelination) and myofibers, which are replaced by fibrous adipose tissue. 13 14 15 Studies on autopsy tissues of type-1 SMA patients suggested impairments in the radial growth of motor axons during mid-gestation. 13 This early loss of motor neurons corresponds to high levels of cerebrospinal fluid and blood neurofilaments, which are neuronal-specific cytoskeletal proteins released from neurons during degeneration. All of this evidence suggests that the disease starts very early, in utero, before the onset of clear clinical signs. 16

### Clinical classification of SMA

The disease has a phenotypic spectrum classified by age at onset and maximum motor milestone achieved. More than half of the patients present the severe phenotype of type-1 SMA, with onset of symptoms within the first 6 months of age, with a “floppy infant” presentation. These infants fail to achieve the free-sitting milestone, and, without drug treatment and ventilator support, they may experience death in early infancy, with a life expectancy lower than 2 years. Type-2 SMA has a milder course, with onset of symptoms between 6 and 18 months of age. Per definition, these patients do manage free sitting, but not independent walking. The latter is achieved in patients with type-3 SMA, in whom symptom onset occurs during infancy or adolescence. They achieve independent walking, although they usually lose this ability throughout their lives. In addition, some classifications define type-0 and type-4 SMA, with prenatal onset or a very mild phenothype respectively. 17 18 19

### Diagnostic tests are available and accurate

Electromyography and muscle biopsy are no longer routinely performed in cases of suspected SMA, for they were gradually replaced by genetic testing. SMN1 deletions and the *SMN2* copy number can be determined by quantitative real-time polymerase chain reaction (PCR), multiplex ligation-dependent probe amplification (MLPA), digital PCR, and next-generation sequencing techniques. For the quantification of the *SMN2* copy number, MLPA is considered the gold standard. Newborn screening identifies ∼ 95% of patients with SMA through the MLPA test, although it is not able to identify those with a point mutation on 1 of the alleles, which requires gene sequencing techniques, additionally. After genetic confirmation of the disease, parents should receive genetic counseling to receive explanations on the genetic basis of SMA and the risk of recurrence in their children. 20 Genetic counseling must be carried out by professionals with specific training, such as genetic counselors or medical geneticists, and must also be provided to all carriers identified by the NBS.

### Early treatment trials

New therapies (with nusinersen, onasemnogene abeparvovec, and risdiplam), which can dramatically modify the natural history of SMA, have been studied in different clinical trials as well as in the real world.

Many clinical trials have shown that early treatment of SMA is crucial to maximize the therapeutic effects. Approximately 30 to 60% (depending on the stage of treatment initiation and the patient's baseline functional status) of children with type-1 SMA treated after the onset of disease manifestations achieve the ability to sit independently, and only individual patients acquire the ability to walk with help. The chance of achieving a new milestone is lower the later the treatment starts. In conclusion, if any treatment is started when signs or symptoms of the disease are already present, the benefits obtained from the medications are not the same, and all patients will present some type of impairment. 21

On the other hand, clinical trials with presymptomatic patients 22 23 have shown better results, with individuals developing as normal children regarding motor development or having mild symptoms of the disease. Table 1 summarizes the presymptomatic clinical trials that have been carried out. Therefore, every effort should be made to diagnose and to treat SMA in the presymptomatic period.

**Table 1:** Clinical trials with “presymptomatic”* spinal muscular atrophy patients 4

Clinical trial number/therapy	Subjects	Endpoint	Publication
NCT02386553/Nusinersen	Participants: 25; 2 *SMN2* copies = 15; 3 *SMN2* copies = 10	Time until death or respiratory intervention; Motor function, and safety	De Vivo et al. (2019) 22 Crawford et al. (2023) 23
NCT03505099/Onasemnogene abeparvovec	Participants: 29 Two *SMN2* copies = 14 Three *SMN2* copies =15	Achieve sitting alone; 5 seconds/30 seconds; Survival; Walking alone	Strauss et al. (2022) 24 Strauss et al. (2022) 25
NCT03779334/Risdiplam	Participants: 25 Two *SMN2* copies Three *SMN2* copies	Sitting without support; Manifesting clinical signs; Time until death or permanent ventilation; Motor milestones	Finkel et al. (2023) 26

Note: *Absence of clinical signs or symptoms during screening.

### Therapeutic window in SMA patient not identified by symptoms (NIS)

The results from clinical trials conducted in presymptomatic babies cannot apply to the overall population of children identified by NBS, as the outcomes for patients who become symptomatic before treatment are much less favorable than the outcomes for those treated before clinically-manifested SMA. That difference in outcomes confirms the urgency for rapid treatment of infants diagnosed with SMA, especially those with two copies of *SMN2* . 5

A systematic review 5 of articles published up to January 2023 on the evolution of patients after NBS for SMA presented the results of 153 patients with 2 or 3 copies of *SMN2* , and the treatment was started at the mean ages of 23 and 52 days respectively. All of the patients, except for 1, with 3 copies of *SMN2* treated before the age of 42 days presented with normal motor development. Half of the patients with 2 copies of *SMN2* presented symptoms of SMA at the 1st treatment. Of the subjects treated “presymptomatically”, 61% showed no motor delay at a mean age of 15 months, but 36% presented clear motor impairment. Among the patients with 2 copies of *SMN2* , 8% had nutritional support and 12% used non-invasive ventilation at the last follow-up. 5 These data indicate the urgency of starting treatment as soon as possible, ideally within the first 14 days of life, especially in individuals with 2 copies of *SMN2* .

Even in patients treated “presymptomatically” different outcomes may be observed. While nearly all of those with 3 copies of *SMN2* demonstrate normal development, 1/3 of the patients with 2 copies of *SMN2* still show motor delays, and some present mild feeding and respiratory impairments. 24 25 Thus, the term *presymptomatic* needs to be better understood, since “presymptomatic” does not mean that these patients do not present motor neuron degeneration or signs of the disease.

Studies 27 show that some patients, even without muscle weakness or hypotonia, may present some clinical signs of the disease, such as absence of reflexes, fasciculations, or compound muscle action potential (CMAP) amplitude ≤ 2 mV. Unfortunately, these patients will not progress as well with treatment, and some motor impairment can be expected. 27 These data reinforce what the authors 27 refer to as “urgency neurogenetics”, with the need to treat “presymptomatic” SMA patients as soon as possible because of the rapid loss of motor neurons in the first weeks of life.

Recently, swallowing abnormalities presenting as early as 3 months of age have been described in patients treated in the presymptomatic stage, who need to be carefully managed. 28 Also, considering the cognitive development, they can present lower cognitive scores, which are related to the lower number of *SMN2* copies. 29

**Figure 1:**
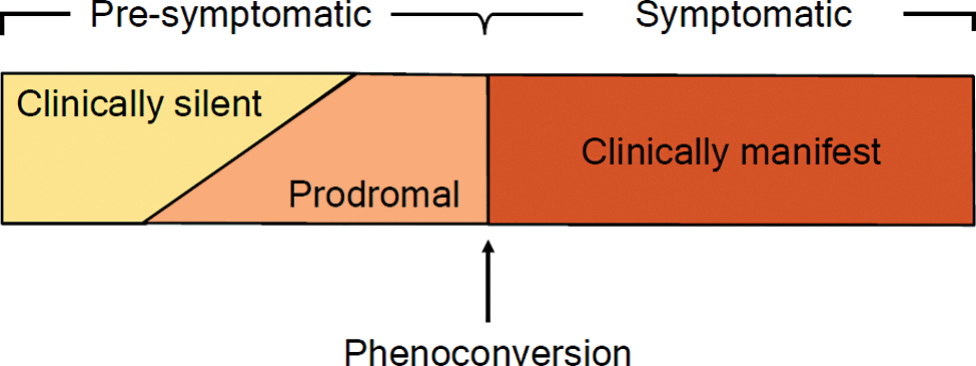
Concept of phenoconversion for spinal muscular atrophy (SMA) patients identified by newborn screening (NBS). 30

There are many gaps in our understanding of early and “presymptomatic” SMA. In 2022, Finkel and Benatar 30 proposed the concept of *phenoconversion* for SMA patients identified by NBS programs: the “presymptomatic” stage includes patients in the clinically-silent and prodromal stages who, later, will convert to the symptomatic or clinically-manifest stage ( Figure 1 ). Clinically-silent patients are those without symptoms, whose motor examination has been regarded as normal by an experienced pediatric neurologist. Prodromal disease encompasses those individuals who present subtle symptoms and/or findings on examination that are consistent with SMA but are not definitive. The terms *paucisymptomatic* and *oligosymptomatic* have sometimes been used to describe these patients, while *symptomatic SMA* is the term reserved for those individuals with definite clinical findings that are typical of SMA. The authors 30 emphasize that the clinical identification of phenoconversion may be difficult, and they have proposed the importance of the use of biomarkers which could identify these stages as well as the timing of phenoconversion, especially in patients with 2 *SMN2* copies.

In conclusion, for an optimal response, the treatment should be introduced when motor neurons are still viable. That is the idea summed up by the expression *time is motoneuron* . 31 Unfortunately, in severe and extremely severe forms, the prenatal SMN deficit is significant, and it is not possible to fully restore physiological processes after birth.

### The treatment after NBS

The current consensus is that all patients screened in the NBS with 2 or 3 *SMN2* copies should be treated, but recommendations regarding the treatment and management of patients with 4 copies of *SMN2* have been a subject of discussion. The therapeutic window for the mild and very mild forms has not yet been defined. 32

In the 1st expert consensus statement, published in 2018, 33 only half of the experts were supportive of early treatment of patients with 4 copies of SMN2; therefore, a wait-and-see strategy was recommended. Two years later, the same working group 34 published a revision in which 11 of the 12 experts on the panel recommended the early treatment of children with 4 copies of *SMN2* . Based on the limited available data, it is very likely that early treatment will ensure normal motor development. 5 There is no consensus and little data supporting the treatment of patients with four or more than four copies.

### The timing for treatment is important

In SMA, especially type-1 SMA, the symptoms appear very quickly, and it is not uncommon for presymptomatic babies evaluated at short intervals, such as 1 week, to show clear signs and symptoms of the disease, like hypotonia or atypical diaphragmatic breathing pattern. As long as there are no markers that could differentiate early progressors from later progressors, even a few days can make a difference, especially in patients with 2 copies of *SMN2* . 27

Publications that share experiences from countries with NBS for SMA have warned of the high percentage of patients with 2 copies of *SMN2* already showing symptoms of the disease at the time of screening, raising the question that even NBS is too late for patients with 2 copies. For example, the rate of patients with 2 copies of *SMN2* in the NBS who already had symptoms of the disease reached 40% in Italy 35 and 47% 36 in Germany. 36

In Germany, 37 out of 21 SMA patients identified in NBS projects, only 12 (57%) presented completely-normal development, reaching motor milestones in time and having no bulbar or respiratory problems. Two patients (9.5%) needed feeding via gastric tube, and 1 patient (4.8%) had respiratory problems. Children with baseline CMAP amplitude higher than 3 mV developed normally, reached milestones within the normal age range, and did not show any symptoms of SMA. The presence or absence of deep tendon reflexes did not show a correlation with the outcome in these patients. 37

Therefore, the start of treatment in patients who underwent NBS must be immediate, being considered a neurological emergency, especially in newborns with 2 copies of SMN2.

### Economic burden of SMA and NBS impact

Cost and quality of life studies 38 indicate that SMA has a significant impact on the daily lives of patients and their families, as well as on society, which bears the cost of health care. The annual costs involving type-1 SMA range from $75,047 to $196,429, not including the costs of disease-modifying drug. The annual costs of late-onset SMA of types 2, 3, and 4, which are often pooled in health care cost estimates, range from $27,157 to $82,474. 38

Previous studies 38 39 40 have investigated the costs associated with the SMA treatment and the differences in costs depending on the stage in which the treatment begins. The overall costs were lower for patients who received treatment after early testing compared to those who were treated after presenting with symptoms. 39 40 This highlights the importance of early patient identification and treatment in reducing the total societal costs of SMA, particularly where treatments are available for both presymptomatic and postsymptomatic patients.

Six medico-economic analyses have evaluated all the data on lifetime and have shown that SMA NBS, when coupled with early treatment, is cost-effective compared to late treatment following clinical diagnosis. 39 40 41 42 43 44

## NEWBORN SCREENING

### What criteria are used to include a disease in the NBS?

In general, the criteria commonly used in screening programs follow those proposed by James Wilson and Gunnar Jungner in 1968, 45 and are recommended by the World Health Organization (WHO): 46

- The natural history of the disease must be well known;

- It is possible to identify the disease before the onset of clinical manifestations;

- The possibility of treatment at an early stage should bring greater benefits, compared to treatment after the clinical manifestation of the disease;

- Existence of an adequate test for diagnosis at an early stage, capable of being incorporated into routines for diagnosing other diseases already incorporated in neonatal screening tests;

- The incidence of the disease must be high in the population;

- The cost-to-benefit ratio of population screening must be considered as well as its effectiveness; and

- There must be broad acceptance by the population.

### NBS for SMA around the world

The rapid approval of multiple treatment options for SMA over a short period has excited the community and challenged providers to implement new standard-of-care models. Prior to the approvals of the new therapies for SMA, the treatment paradigms had evolved from a primarily palliative or reactive approach to a more proactive care model with attention to a multitude of needs, including respiratory, nutritional, and orthopedic needs. 27 Consensus statements on the standard of care, initially published in 2007 and revised in 2018, reflect these changes. 47 48 49 The effort to include SMA in the NBS increased after the approval of nusinersen and due to the growing evidence of better outcomes in patients with earlier treatment. 22 At the moment, one of the most significant developments in the new models of care for SMA is the implementation of NBS, which has been carried out in an increasing number of countries. In 2018, the Advisory Committee on Heritable Disorders and Genetic Diseases in Newborn and Children (ACHDNC), which is part of the United States Department of Health and Human Services, recommended the inclusion of SMA on the Recommended Uniform Screening Panel (RUSP), and several American states have implemented NBS programs for SMA, actually covering 100% of infants born in the United States. 50 In Europe, the SMA Newborn Screening Alliance was created with the aim of implementing neonatal screening for SMA across all European countries by 2025. 51

In this context, NBS programs for SMA have been implemented in Taiwan, the United States, Belgium, Germany, Italy, Australia, Canada, Japan, and, recently, Ukraine. 5 21 35 36 52 53 54 55 56 57 58 59 60 61 62

**Figure 2:**
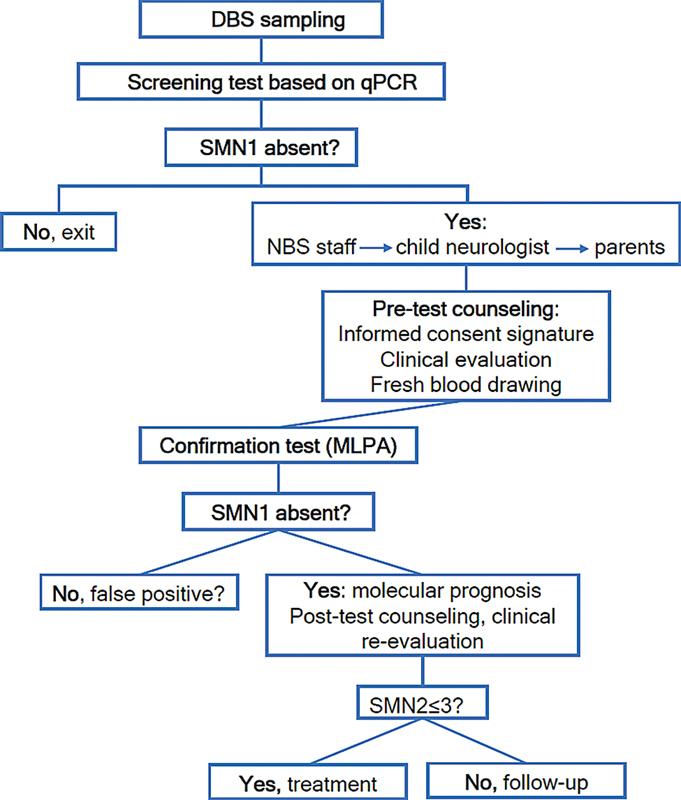
Workflow for the NBS program for SMA (modified by Abiusi et al., 2023). 35

Screening tests for SMA commonly involve the extraction of DNA from samples of dried blood spot (DBS) and the use of real-time quantitative PCR to determine the presence or absence of the responsible gene, *SMN1* , although PCR and digital PCR are also used. 54 The diagnosis of SMA is confirmed based on the results of the MLPA assay using a newly-collected blood sample ( Figure 2 ). In Table 2 , information about NBS for SMA in various countries is summarized.

**Table 2:** Details on NBS for SMA in different countries

Region	Countries	Starting year	% NB Screened
**Americas**	United States (P) Canada (P)	2016 2020	61–70% 31–40%
**Europe**	Germany (P) Belgium (P) Italy (P) Russia (P)	2018 2018 2019 2019	11–20% 45% 11–20% <10%
**Asia**	Taiwan (W) Japan (P)	2014 2020	81–90% < 10%
**Oceania**	Australia (P)	2018	21–40%

Abbreviations: NB, newborn; NBS, newborn screening; P, part of country; SMA, spinal muscular atrophy; W, whole country.

Source: Dangouloff et al. (2021). 54

Although rapid advances in therapeutics and genetic screening technologies have driven recommendations for pilot NBS for SMA programs in select jurisdictions, the implementation of this type of screening continues to lag globally. As of 2021, only 9 countries had implemented NBS for SMA, with fewer than 2% of newborns across the world screened for the condition. 54

### NBS in Brazil

In Brazil, the NBS started in 1976 through isolated initiatives. The creation of the National NBS Program (Programa Nacional de Triagem Neonatal, in Portuguese) in 2001 has provided a new perspective in the public health system, 63 which was implemented in all states by their Health Departments. Currently, the Brazilian NBS program includes phenylketonuria, congenital hypothyroidism, sickle cell disease/other hemoglobinopathies, cystic fibrosis, congenital adrenal hyperplasia, and biotinidase deficiency. The blood test is collected between the third and fifth days of life at primary health care settings or maternity hospitals. 64

According to the NBS indicators made available by the Brazilian Ministry of Health, 65 although the national coverage rate was of 82.53% in 2020, if we consider tests collected up to the fifth day of life, the coverage drops to 58.3% in the same year, and the median age of the newborn in days on the date of the first medical appointment varied between 30 and 54 days. 65

In May 2021, the Brazilian Congress enacted a law (no. 14,154) that expanded the screening from 6 to more than 50 diseases in 5 steps, including SMA, which should be part of Brazilian NBS shortly. The steps published in this document are: 66

- Step 1: phenylketonuria and other hyperphenylalaninemia, congenital hypothyroidism, sickle cell disease and other hemoglobinopathies, cystic fibrosis, congenital adrenal hyperplasia, biotinidase deficiency, congenital toxoplasmosis;

- Step 2: galactosemics, aminoacidopathies, urea cycle disorders, fatty acid beta oxidation disorders;

- Step 3: lysosomal diseases;

- Step 4: primary immunodeficiencies; and

- Step 5: SMA 5q.

Currently, however, not all Brazilian states are in the same step of the NBS. Most of states are in step 1 and some of them are including new diseases in their NBS, in different sequences, based on their realities. To date, only the Federal District of Brasília and 1 out of the 26 Brazilian states (Minas Gerais) have already included SMA in the NBS, and 2 pilot studies have been conducted. 67

### What could be the challenges of NBS for SMA?

Several countries that have implemented NBS for SMA have initiated the program through pilot studies, like the United States, Germany, Belgium, Italy, and Australia. While in these pilot studies a single laboratory performed the NBS using its own well-established method and the notification and follow-up care of the families was provided by few well-connected centers, transition to a nationwide NBS program could bring some new challenges. With appropriate preparation, it is possible to extend NBS without significant losses in speed or quality. 1

Dangouloff et al. 54 reported the main obstacles encountered during the implementation of NBS for SMA: lack of financial resources (55–68%), organizational issues (33–21%), lack of equipment (22–29%), lack of government support (44–30%), and lack of human resources (11–29%). Moreover, they described the measures that could support NBS for SMA: resources and support of the government (66–67%), clear professional consensus at a national level (22–32%), cost-benefit analysis (55–70%), long-term follow-up data on the treatment of presymptomatic patients (55–53%), and assistance with implementation practicalities. 54

In Brazil, many of these obstacles and measures could be quite similar. However, given the size of the country, we need to understand that needs could be variable in different regions. Currently, the Brazilian NBS has different levels of coverage and steps of disease screening because of this heterogeneity, and the inclusion of SMA 5q in the NBS should be planned and organized respecting these regional peculiarities. However, the main problems we should consider in Brazil are:

- The *SMN2* copy number assessment: it requires quantitative methodologies that are not easily implemented in most laboratories. Along these lines, surprising discordance among laboratories (of around 40%) has been reported. The development of shared guidelines to optimize and standardize the reliability of *SMN2* copy number assessment among referral laboratories is very important, as well as strengthening of the *SMN2* collaborative networks between laboratory and clinical settings before NBS implementation. 68

- Time required to receive the result of the screening test: it can be variable based on the local organization for collection of the sample and laboratory structure;

- Time required to schedule the first appointment: it can be variable based on the local organization in terms of contacting the positive patients and laboratory structure. At this stage, parents will be notified about the result and significance of the screening test, they will receive information about the disease, and the baby will be clinically evaluated. A second sample will be collected for confirmatory testing;

- Time required to receive the result of the confirmatory test: it can be variable based on the local organization and laboratory structure;

- Time required to schedule the second appointment: it can also be variable based on the local conditions, availability of the therapies, hospital structure and environment conditions. At this stage, the parents will be informed about the result of confirmatory test and about the treatment based in the new therapies to support the decision of which therapy to prescribe;

- Who will be treated and with which medication: although most countries currently still treat babies screened with 1 to 3 copies of the *SMN2* gene, there is a tendency to include patients with 4 copies. It is important that this topic be discussed with experts and defined as soon as possible, as in the Federal District of Brasília and in the state of Minas Gerais screening for SMA has already started. The choice of medication choice should be based on the Clinical Protocols and Therapeutic Guidelines (Protocolos Clínicos e Diretrizes Terapêuticas, PCDT, in Portuguese) of the National Committee for the Inclusion of Technology within the Brazilian Unified Health System (Comissão Nacional de Incorporação de Tecnologias no Sistema Único de Saúde, CONITEC, in Portuguese). 69 Nevertheless, the faster the medication is started, the better the prognosis;

- Maintenance of the treatment based on the therapy chosen: we should consider the regular supply of therapies that are continuous based on environmental and economic conditions; and

- Follow-up of the patients: we should consider the availability of multidisciplinary teams in the follow-up of SMA patients and environmental conditions.

In conclusion, the diagnostic journey in the case of a rare disorder is usually very long and hard, especially considering that SMA is a neurodegenerative disorder with a catastrophic natural history. In order to achieve the best therapeutic window in SMA, the diagnostic delay can be overcome with a well-established SMA NBS program, which could yield remarkable therapeutic benefits for the patients and their families, even if it is established with limited financial funds in a more cost-effective way.

The Brazilian NBS needs to be developed based on the conditions of each region, but it is also necessary to understand the importance of starting the treatment as soon as possible, ideally until 14 days of life, especially for patients with 2 *SMN2* copies. Implementing screening without ensuring a complete program with access to early diagnosis and treatment for asymptomatic babies would not only be expensive for the health system but mainly catastrophic for babies and their families.

Expectations regarding the efficacy of the treatment efficacy of screened babies must be aligned as soon as possible between parents and pediatricians, who should expalin that some patients could present some motor delay or signs of weakness. It is extremely important that SMA patients can be clinically monitored and treated in specialized reference centers.
